# Paraneoplastic Erythroderma: An Unprecedented Initial Manifestation of In Situ Follicular Neoplasia

**DOI:** 10.7759/cureus.99873

**Published:** 2025-12-22

**Authors:** Manjul Srivastava

**Affiliations:** 1 Internal Medicine, Northwest Medical Center, Tucson, USA

**Keywords:** follicular, in-situ, lymphoma, neoplasia, paraneoplastic erythroderma

## Abstract

Paraneoplastic erythroderma is a severe cutaneous reaction characterized by diffuse erythema and scaling of the body surface area. While typically associated with hematologic malignancies such as cutaneous T-cell lymphoma or solid tumors, it is notoriously refractory to standard dermatologic therapies. *In situ* follicular neoplasia (ISFN) is generally regarded as an indolent, clinically silent condition discovered incidentally. We present a unique case of severe, treatment-resistant erythroderma in an elderly male, which resolved completely following the excision of lymph nodes positive for ISFN. To our knowledge, this is the first reported association between ISFN and paraneoplastic erythroderma, challenging the paradigm that ISFN is a biologically inert entity.

## Introduction

Paraneoplastic erythroderma can be the earliest or sole manifestation of an occult internal malignancy, developing in the absence of direct tumor cell infiltration of the skin. It is a severe cutaneous reaction syndrome characterized by diffuse erythema and scaling involving at least 90% of the body surface area [1]. This condition, often distinct to hematologic malignancies (e.g., cutaneous T-cell lymphoma) and solid tumors, proves refractory to standard skin regimens; clinical resolution is typically contingent upon successful management of the primary cancer. Notably, the skin in paraneoplastic erythroderma shows no histopathologic evidence of direct tumor infiltration; instead, the cutaneous findings are thought to arise from cytokine-mediated or immune-dysregulated mechanisms triggered by the underlying cancer.

*In situ* follicular neoplasia (ISFN) is generally regarded as an indolent, clinically silent precursor lesion within the spectrum of follicular lymphoid neoplasms. It is most often discovered incidentally during a lymph node biopsy performed for unrelated clinical indications. Thus, ISFN represents a biologically early and localized neoplastic process with minimal clinical impact at presentation, but one that carries potential, albeit low risk for progression. Recognition of this entity is essential to guide appropriate work-up, avoid overdiagnosis, and ensure long-term clinical surveillance.

This is a unique case of severe, treatment-resistant erythroderma in an elderly male, which resolved completely following the excision of lymph nodes positive for ISFN. 

## Case presentation

History and physical examination

**Figure 1:**
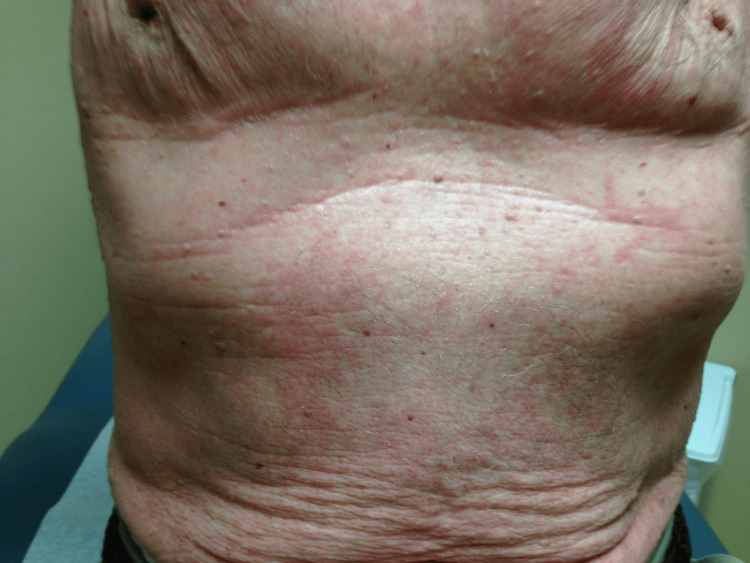
Intense pruritic rash over the chest.

An 86-year-old male presented with a four-month history of a progressive, intensely pruritic rash. His medical history included hyperlipidemia, gastroesophageal reflux disease (GERD), treated pulmonary coccidioidomycosis, and osteopenia. The eruption originated on the upper extremities before generalizing to involve the entire body (Figure 1).

Physical examination revealed a distressed patient with shivering despite normal room temperature. Vital signs showed a temperature of 36.9°C, heart rate of 80 bpm, and blood pressure of 115/70 mmHg, respiratory rate of 14 breaths per minute, and an oxygen saturation of 94%. Dermatologic assessment confirmed generalized, confluent erythema with overlying scale consistent with erythroderma.

**Figure 2:**
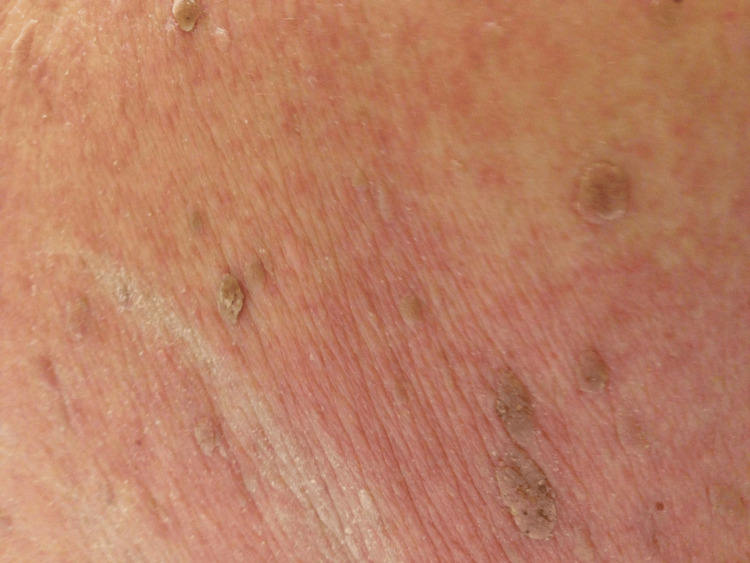
Multiple new seborrheic keratosis on the erythematous skin.

Over a period of one month, widespread eruption of hyperpigmented, warty papules and plaques (seborrheic keratosis) developed on the patient's back and abdomen (Figure 2).

**Figure 3:**
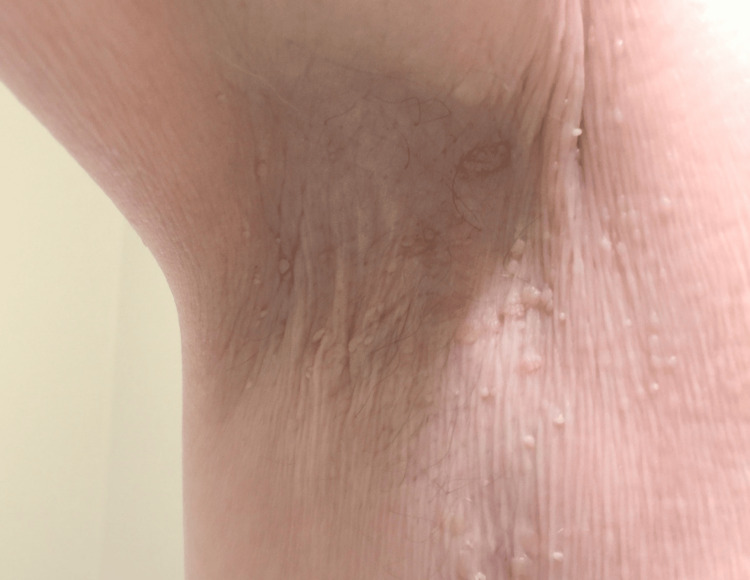
Skin growth in arm pit.

Additionally, hyperkeratotic, papillomatous plaques were noted in the bilateral axillae (Figure 3).

Initial management with high-potency topical steroids yielded no relief. The patient's symptoms worsened, and he developed flushing, drenching night sweats, intolerance to temperature, fatigue, loss of appetite and swelling in his legs. There was extreme sensitivity to warm showers, causing rapid heart rate and generalized diaphoresis. The swelling started to spread to his arm, abdominal wall and scrotal area, causing extreme discomfort. His symptoms started getting worse, and he was subsequently hospitalized following an episode of supraventricular tachycardia. Patient was treated with diuretic Lasix 40 mg daily, which was tapered to 20 mg and Metoprolol succinate 50 mg daily.

Diagnostic workup

The patient's laboratory results are shown in Table 1.

**Table 1:** Patient's laboratory results.

Type of labs	Patient lab results	Reference range
White blood count	10,600 /µL	4,500-11,000 /µL
Neutrophils	8,190 /µl	2,500-7,000 /µL
Lymphocytes	1,400 /µL	1,000-4,800 /µL
Monocytes	570 /µ/L	200-800 /µL
Eosinophils	410 /µ/L	Less than 500 /µL
Basophils	60 /µL	0-300 /µL
Hemoglobin	13.7 g/dl	13.5-17.5 g/dl
Hematocrit	43.4%	40.7-50.3%
Mean corpuscular volume (MCV)	100.1 fl	80 -100 fl
Mean corpuscular hemoglobin (MCH)	37.5 g/dl	32-36 g/dl
Red cell distribution width (RDW)	14.2%	11.8-14.5%
Platelet	346,000/ µL	150,000-450,000 /µL
Mean platelet volume	6.5 fl	7.5-11.5 fl
Aspartate aminotransferase	27 IU/L	8-48 IU/L
Alanine transferase	26 IU/L	7-55 IU/L
Alkaline phosphatase	66 IU/L	44-147 IU/L
Blood urea	17 mg/dl	7-20 mg/dl
Creatinine	0.65 mg/dl	0.7-1.3 mg/dl
Glomerular filtration rate (GFR)	88 ml/min/1.73 m^2^	60-89 ml/min/1.73 m^2^
Glucose	85 mg/dl	70-130 mg/dl
Sodium	138 mmol/l	135-145 mmol/l
Potassium	4.1mmol/l	3.5-5 mmol/l
Chloride	103 mmol/L	96-106 mmol/L
Bicarbonate	27 mmol/l	22-29 mmol/l
Calcium	9.4mg/dl	8.6-10.2 mg/dl
Anion gap	8 meq/l	8-12 meq/l
Total protein	6.3 g/dl	6-8.3 g/dl
Albumin	4.2 g/dl	3.5-5.5 g/dl
Globulin	2.1 g/dl	2-3.5 g/dl
Lactic dehydrogenase	265 IU/L	140-280 IU/L
Thyroid-stimulating hormone (TSH)	3.52 mU/L	0.27-4.2 mIU/L
Free thyroxine (T4)	T4 0.8ng/dL	0.8-1.8 ng/dl
Sediment rate	11 mm/hour	Less than 20 mm/hour
C-reactive protein (CRP)	42.3 mg/L	< 1.0 mg/dl
Creatine phospho-kinase (CPK)	199 IU/L	10-120 IU/L
Antinuclear antibody (ANA)	negative	negative
Rheumatoid factor	9 I U/ml	Less than 20 IU/ml
Serum tryptase	11.4 ng/ml	0-15 ng/ml
24 hour urine total free metanephrine and nonmetanephrine	174 pg/ml	< 205 pg/ml

Skin biopsy from left waist, upper chest, right lateral abdomen and right anterior thigh showed no evidence for dermatomyositis, other connective tissue disorder, mycosis fungoid or primary cutaneous malignancy.

**Figure 4:**
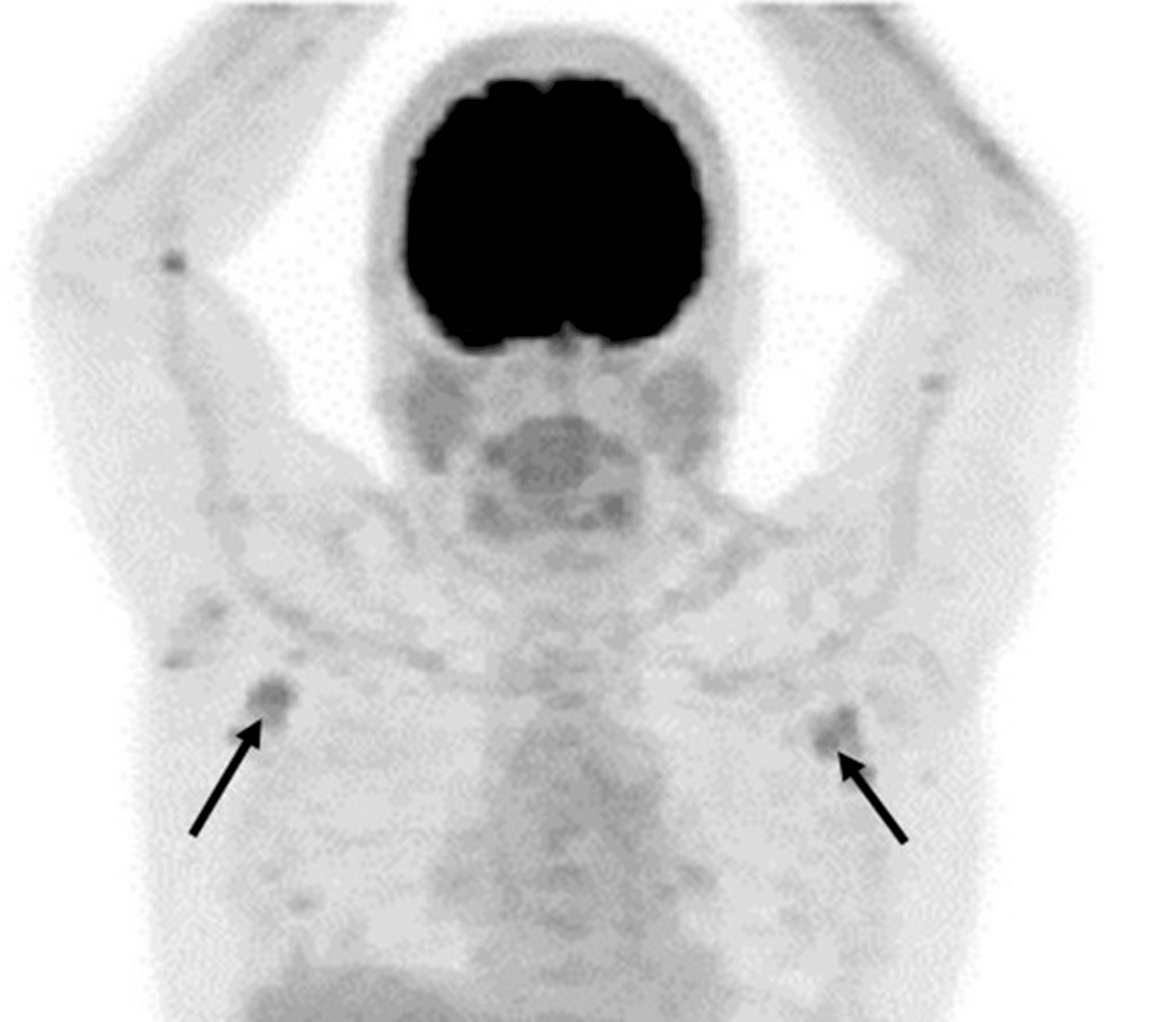
. Axillary lymph nodes (arrows) on PET scan image.

Due to his new onset of multiple seborrheic keratosis and concerns of Leser-Trelat-like symptoms malignancy workup was done. Endoscopy and colonoscopy were negative for malignancy, except for a 9 mm benign tubulovillous adenoma in proximal ascending colon. CT scans were essentially negative. Workup for carcinoid tumors and pheochromocytoma (including octreotide scan and 24-hour urine metanephrines) was also negative. There was a mild elevation of CPK, but the muscle biopsy was also negative for dermatomyositis. Bone marrow biopsy demonstrated trilinear hematopoiesis with a normal myeloid: erythroid ratio and no morphologic or immunophenotypic evidence of lymphoma. Lymphocytes were not increased, and no morphological or immune phenotypic evidence of lymphoma was found. Mastocytosis was ruled out. After six months of extensive work up PET scan was done, which showed metabolic activity in the right and left axillary lymph nodes; therefore, tissue diagnosis became imperative (Figure 4).

Pathology and diagnosis

**Figure 5:**
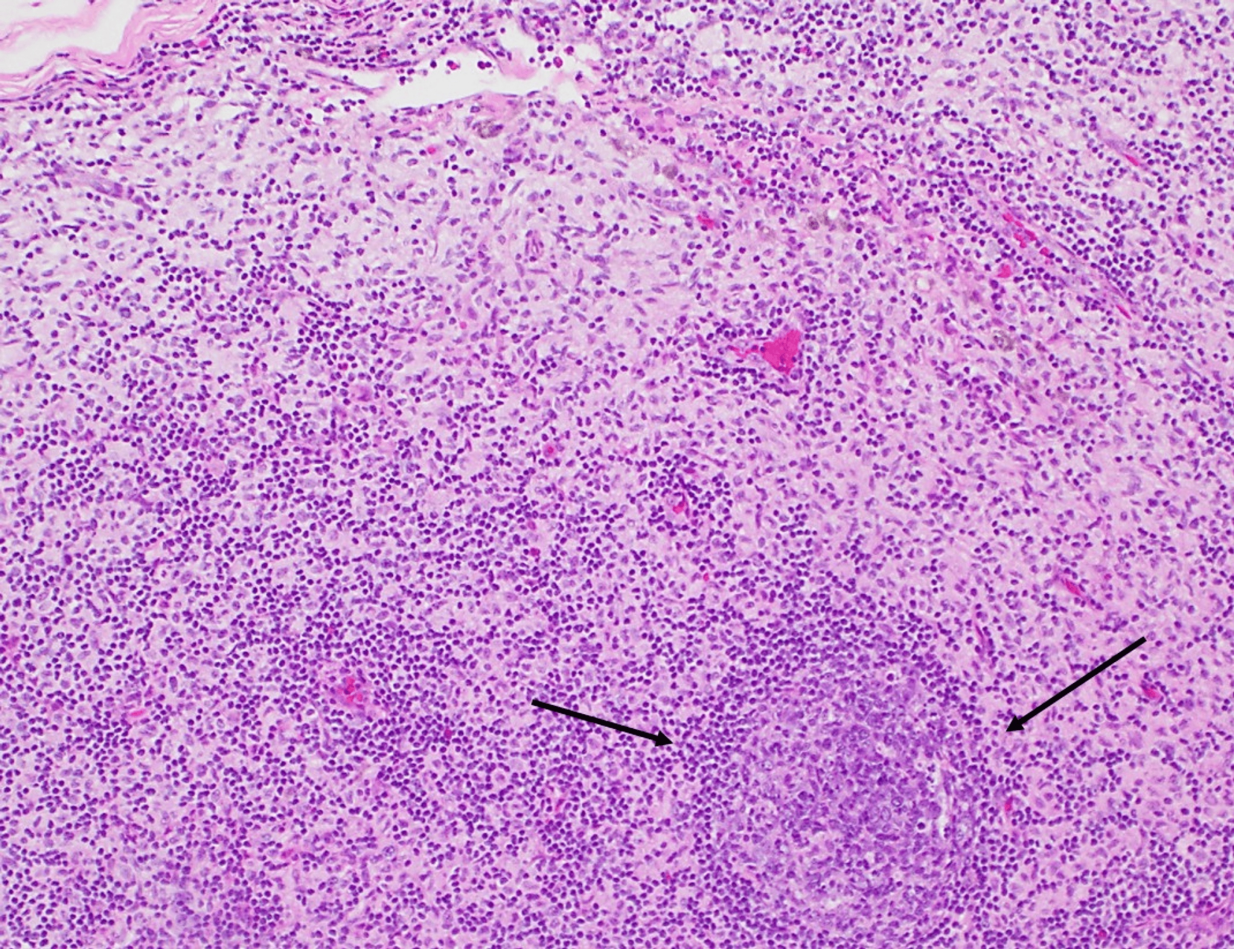
Axillary lymph node, H&E stain, 10x. The follicle architecture and the surrounding mantle is intact. Some follicles showed dark staining of the germinal centers.

Excisional biopsy of the right and left axillary lymph nodes revealed preserved nodal architecture with intact mantle zones (Figure 5). However, focal follicles exhibited dark-staining germinal centers.

**Figure 6:**
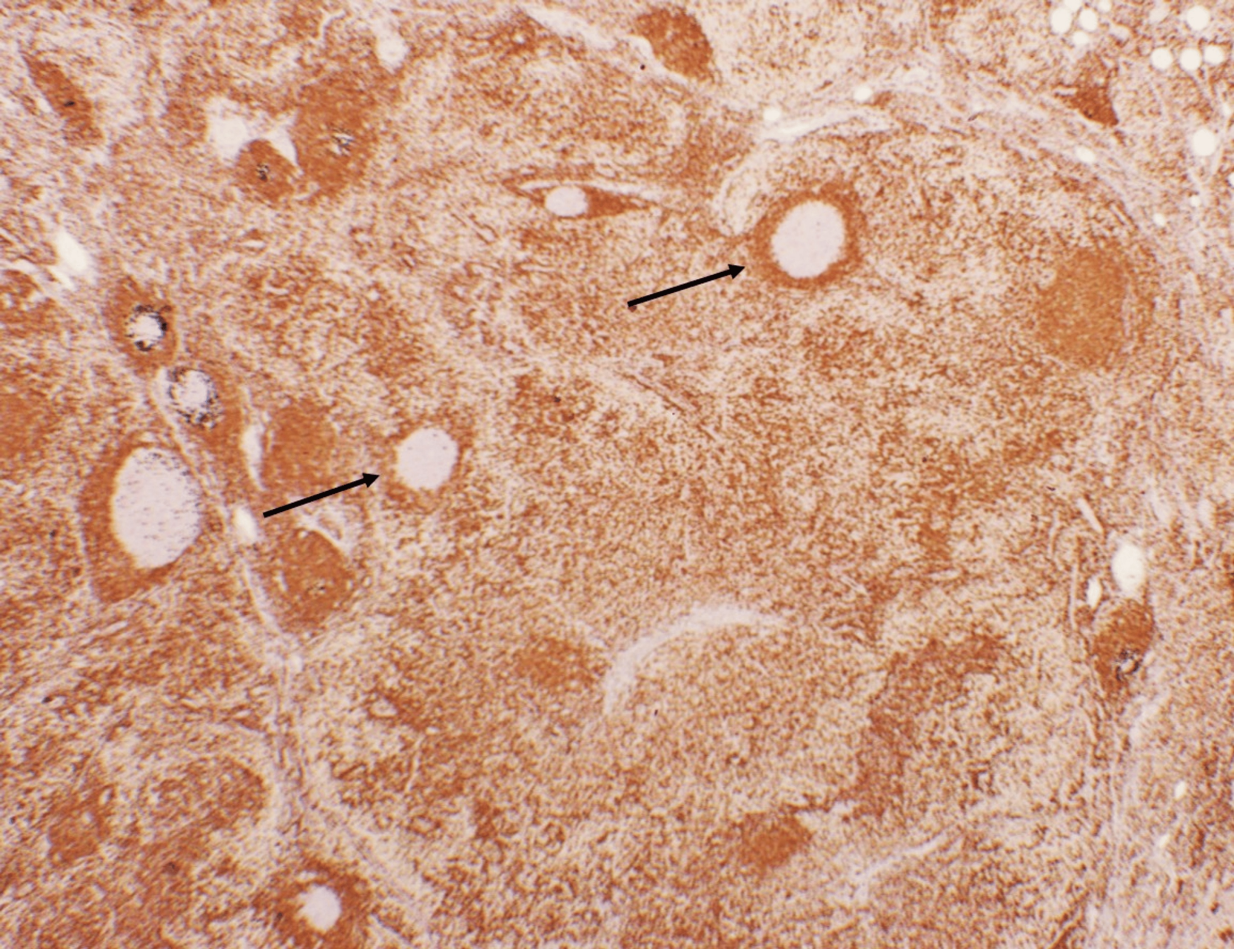
Lymph node showed focal atypical germinal centers with the strong Bcl-2 staining consistent with in situ follicular neoplasia (marked with asterisks) within a background otherwise intact mantle zones.

Immunohistocytometry was strongly positive for Bcl-2, Bcl-6, and CD10 in germinal centers. Figure 6 shows strong Bcl-2 staining of germinal centers, which is characteristic of* in situ* follicular neoplasia. Polymerase chain reaction (PCR) testing for T cell receptor clonality was negative (no evidence of a monoclonal T cell population).

Outcomes

Following the surgical resection of the bilateral axillary lymph nodes, the patient experienced a complete resolution of symptoms within six weeks. The erythroderma, seborrheic keratoses (Leser-Trélat sign), flushing, night sweats, and edema resolved with no recurrence. The patient remains in remission under close clinical surveillance for potential progression to follicular lymphoma.

## Discussion

This represents the first documented instance of ISFN associated with paraneoplastic erythroderma based on a comprehensive literature review. Paraneoplastic erythroderma is a cutaneous manifestation of internal malignancy occurring in the absence of direct tumor infiltration of the skin. It is rare, accounting for approximately 1% of erythroderma cases, and may constitute the earliest or sole sign of an underlying neoplasm [2]. Reported associations most frequently involve solid tumors, including gastric, prostate, and lung cancers, as well as certain hematologic malignancies. T-cell lymphomas such as mycosis fungoides and Sézary syndrome are the classical lymphoid causes; acute myeloid leukemia has also been described. Importantly, the skin in these cases does not show tumor cell infiltration.

Clinically, paraneoplastic erythroderma is characterized by diffuse erythema, intense pruritus, peripheral lymphadenopathy, and impaired thermoregulation [1,3-4]. The condition reflects accelerated epidermal turnover, resulting in erythema and scaling involving more than 90% of the body surface area and marked disruption of barrier function. It may precede or coincide with the diagnosis of malignancy. Skin biopsy typically reveals nonspecific inflammatory changes without evidence of neoplastic involvement. The dermatosis is typically refractory to dermatologic therapies and improves only with effective treatment of the underlying cancer.

**Figure 7:**
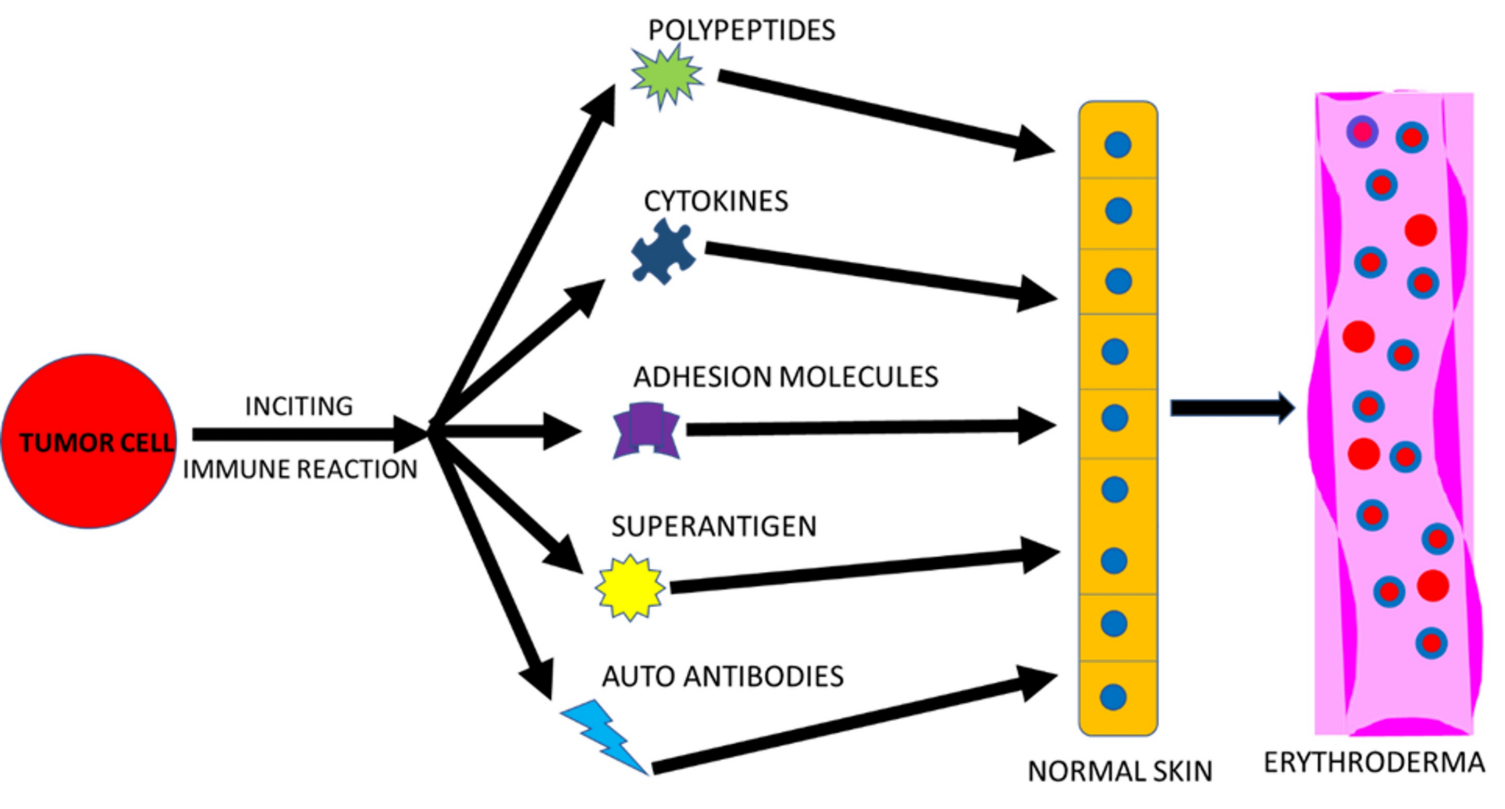
Mechanisms of paraneoplastic erythroderma. This is the original figure created by the author of the manuscript.

The concept of paraneoplastic dermatoses dates back to Hebra in 1868, who first recognized that cutaneous findings may signal visceral malignancy [1,4-5]. In 1986, McLean proposed diagnostic criteria to establish a causal relationship between a dermatosis and an internal malignancy: the dermatosis arises only after development of the malignant neoplasm, and the dermatosis and the neoplasm follow a parallel clinical course, improving with treatment of the cancer and recurring with relapse or metastasis [6]. Mechanistically, lymphocyte homing to the skin, driven by tumor-related immunologic signals, has been suggested to explain cutaneous involvement in some lymphomas [7]. Paraneoplastic processes are thought to be mediated by tumor-associated polypeptides, hormones, cytokines, antibodies, or growth factors (Figure 7), which alter cellular activity and manifest as dermatologic disease [1,5,8-9].

Although paraneoplastic erythroderma is most often described in association with T-cell lymphomas, primary cutaneous presentations of B-cell lymphomas are exceedingly rare, with only isolated cases reported in mantle cell lymphoma and diffuse large B-cell lymphoma [1,3,10]. Non-Hodgkin lymphomas comprise both T-cell and B-cell neoplasms; major B-cell subtypes include diffuse large B-cell lymphoma, follicular lymphoma, marginal zone lymphoma, small lymphocytic lymphoma/chronic lymphocytic leukemia, Burkitt lymphoma, and mantle cell lymphoma. According to the 5th edition of the WHO Classification of haematolymphoid neoplasms, follicular lymphomas encompass several entities, including classic follicular lymphoma, predominantly diffuse follicular lymphoma, follicular large B-cell lymphoma, ISFN, pediatric-type follicular lymphoma, duodenal-type follicular lymphoma, and primary cutaneous follicle-center lymphoma [11].

ISFN is a clonal, noninvasive B-cell proliferation confined to the germinal centers of otherwise reactive lymph nodes. It is typically an incidental finding, most commonly in older adults, and is considered a precursor lesion to follicular lymphoma. First described in 2002 as an early event in follicular lymphomagenesis, the entity was designated as intrafollicular neoplasia by the WHO in 2008 and renamed as *in situ* follicular neoplasia in 2016. ISFN occurs in approximately 2-3% of reactive lymph nodes. The neoplastic B cells strongly express Bcl-2, CD10, and Bcl-6, while the surrounding lymph node architecture shows reactive follicular hyperplasia without interfollicular infiltration [12-14]. Overexpression of Bcl-2, an anti-apoptotic protein, is central to its pathogenesis and facilitates progression toward overt lymphoma [15,16]. Although the precise risk of progression from ISFN to clinically significant lymphoma remains uncertain, cases of subsequent follicular lymphoma have been documented.

Given this potential, all patients with ISFN should undergo comprehensive evaluation, including physical examination, blood studies, flow cytometry, and CT imaging of the neck, chest, abdomen, and pelvis, and be followed closely. Evidence-based management guidelines are still evolving, but early identification allows appropriate surveillance and timely intervention.

The coexistence of ISFN and paraneoplastic erythroderma represents a particularly uncommon clinical presentation. Because paraneoplastic erythroderma may precede the identification of an underlying hematologic malignancy, clinicians should maintain a high index of suspicion when evaluating unexplained erythroderma that is refractory to standard therapy. Awareness of this complex interplay between dermatologic manifestations and neoplastic processes is essential. Prompt recognition facilitates early diagnosis and treatment of the underlying malignancy, which remains the cornerstone for resolution of the paraneoplastic dermatosis.

## Conclusions

To summarize, this case report demonstrates that even a silent and inert condition like in situ follicular neoplasia can trigger a profound systemic inflammatory response leading to paraneoplastic erythroderma. For clinicians, this is a valuable lesson, highlighting a potential rare cause for unexplained, treatment-resistant erythroderma. A noninvasive neoplasm doesn’t directly invade the skin but can cause erythroderma through immune mediators. Physicians should be aware of this complex interaction between the skin manifestations and neoplastic processes. Early diagnosis is critical to treating the underlying malignancy and resolving the paraneoplastic erythroderma as soon as possible.
